# Ultrasonic Assessment of Optic Nerve Sheath Diameter in Patients at Risk of Sepsis-Associated Brain Dysfunction: A Preliminary Report

**DOI:** 10.3390/ijerph17103656

**Published:** 2020-05-22

**Authors:** Piotr F. Czempik, Jakub Gąsiorek, Aleksandra Bąk, Łukasz J. Krzych

**Affiliations:** 1 Department of Anaesthesiology and Intensive Care, Faculty of Medical Sciences in Katowice, Medical University of Silesia, 40-752 Katowice, Poland; lkrzych@sum.edu.pl; 2 Students’ Scientific Society, Department of Anaesthesiology and Intensive Care, Faculty of Medical Sciences in Katowice, Medical University of Silesia, 40-752 Katowice, Poland; gjakub93@gmail.com (J.G.); aleksandra.bakk@gmail.com (A.B.)

**Keywords:** intracranial pressure, optic nerve sheath diameter, sepsis-associated brain dysfunction, septic shock, ultrasound

## Abstract

Sepsis-associated brain dysfunction (SABD) with increased intracranial pressure (ICP) is a complex pathology that can lead to unfavorable outcome. Ultrasonographic measurement of optic nerve sheath diameter (ONSD) is used for non-invasive assessment of ICP. We aimed to assess the role of ONSD as a SABD screening tool. This prospective preliminary study covered 10 septic shock patients (5 men; aged 65, IQR 50–78 years). ONSD was measured bilaterally from day 1 to 10 (*n* = 1), until discharge (*n* = 3) or death (*n* = 6). The upper limit for ONSD was set at 5.7 mm. Sequential organ failure assessment score was calculated on a daily basis as a surrogate formulti-organ failure due to sepsis in the study population. On day 1, the medians of right and left ONSD were 5.56 (IQR 5.35–6.30) mm and 5.68 (IQR 5.50–6.10) mm, respectively, and four subjects had bilaterally elevated ONSD. Forty-nine out of 80 total measurements performed (61%) exceeded 5.7 mm during the study period. We found no correlations between ONSD and sequential organ failure assessment (SOFA) during the study period (right: R = −0.13–0.63; left R = −0.24–0.63). ONSD measurement should be applied for screening of SABD cautiously. Further research is needed to investigate the exact role of this non-invasive method in the assessment of brain dysfunction in these patients.

## 1. Introduction

Although direct measurement of intracranial pressure (ICP) using an intra-ventricular catheter remains the gold standard, it is burdened with potential serious complications due to its invasiveness [1]. Ultrasonic measurement of optic nerve sheath diameter (ONSD) is a non-invasive method for ICP monitoring. The optic nerve sheath is a direct anatomical extension of the cranial dura mater. Anatomical continuity means that cerebrospinal fluid flows freely between subarachnoid space located around the optic nerve and intra-cranially. An increase in pressure within the intracranial compartment causes swelling of the optic nerve (e.g., papilledema) and is reflected by increased ONSD [2,3].

Septic shock is life-threatening organ dysfunction caused by a dysregulated host response to infection in which circulatory and cellular/metabolic abnormalities lead to increased mortality compared to a simple sepsis [4,5]. The brain is particularly vulnerable in the course of sepsis. Sepsis-associated brain dysfunction (SABD) is a diffuse brain dysfunction due to infection outside the central nervous system. Diagnosis of SABD requires exclusion of central nervous system infection and other possible causes of brain dysfunction. SABD might be present in up to 70% of septic patients [6]. SABD might represent the most frequent organ insufficiency associated with sepsis [7]. Its pathophysiology remains unclear, with potential culprits being neuro-inflammation, excitotoxicity, impaired cerebral autoregulation, and cerebral ischemia [8,9,10]. Pathophysiological changes in SABD may potentially lead to an increase in ICP. Intracranial hypertension may in turn decrease cerebral perfusion and lead to brain damage and compromised outcome in this population of patients [11]. Taking these into consideration, screening for SABD is crucial for early diagnosis and management, especially when mechanical ventilation requires the introduction of sedative agents that preclude neurological assessment. For diagnosis of SABD biomarkers, electrophysiological (electroencephalogram, EEG; evoked potentials) and imaging (computed tomography, magnetic resonance) techniques have been used. These methods may have long turn-around time (send-out biomarker tests), require significant expertise or transport of unstable patients outside the intensive care unit (ICU). The ONSD examination is quick, noninvasive, easy to learn, does not require special equipment, and is performed at the bedside. The use of ONSD as a screening test for non-traumatic cerebral edema in the heterogenous population of patients with unexplained coma in the ICU have been described before. The reasons for coma in the reported study were both primary (cerebrovascular accident, malignancy, infection) and secondary (septic encephalopathy, metabolic encephalopathy, hepatic encephalopathy, anoxic brain injury) brain injury [12]. To our knowledge, the use of ONSD for monitoring SABD specifically has not been described before. The rationale for using ONSD in this population of patients are mentioned above.

Therefore, in this preliminary study, we sought to analyze ONSD in septic shock patients and its potential role in screening for SABD.

## 2. Materials and Methods

This prospective preliminary study covered 10 consecutive septic shock patients, who required mechanical ventilation and sedation, hospitalized in a mixed ICU between January and March 2019. We excluded intracranial pathology based on clinical history and neurologic examination performed before introduction of sedation. All study subjects were placed in the supine position and were mechanically ventilated from admission till resolution of respiratory failure. We decided not to record ventilation status and settings of the ventilator. For diagnosis of septic shock, we applied the third international definition and appropriate diagnostic criteria [4,5]. Demographic, clinical and laboratory data were recorded. General severity of disease was estimated using the following classification systems: acute physiology and chronic health evaluation II (APACHE II), simplified acute physiology score II (SAPS II) and were calculated on admission to the ICU as per national healthcare provider regulations, whereas SOFA was calculated on a daily basis. The SOFA scale represents surrogate for severity of organ dysfunction due to sepsis. It is easily calculated on a daily basis in the ICU and shows patient trajectory during sepsis, and as such, it was decided to examine association between ONSD (a potential surrogate for SABD) and SOFA in the study population during the study period.

Additionally, we also analyzed associations between ONSD and concentration of C-reactive protein (CRP) and lactates. ONSD was measured bilaterally every 24 hours in the late afternoon or evening by a single examiner skilled in the technique. The persons performing the ultrasound examination were aware of the purpose of the study. ONSD measurements were performed for up to 10 consecutive days (*n* = 1) until discharge (*n* = 3) or death (*n* = 6). The measurements were performed with the eyelids closed, using a linear (14.0–6.0 MHz) ultrasound transducer (M7, Mindray, Shenzhen, China), caliper being set at 3 mm posteriorly to the papilla. In order to minimize error, we calculated the mean from three measurement results in each eye. The technique for measuring ONSD have been described before [13], as well as factors having impact on ONSD [14]. Great care was given to avoid excessive pressure on the eyeball. In order to avoid any potential damage to the ocular structures, we followed the ALARA (as low as reasonably achievable) principle and guidelines of the British Medical Ultrasound Society [15,16]. We set the upper limit for ONSD at 5.7 mm [17]. In our ICU, ultrasonic measurement of ONSD is routinely used for monitoring of patients in whom sedation precludes neurological examination. Due to anonymous and non-interventional design of the study, the Bioethics Committee of the Medical University of Silesia in Katowice waived the requirement for informed consent (KNW/0022/KB/200/19).

Statistical analysis was performed using a licensed statistical software (Statistica version 13, StatSoft, Krakow, Poland). Quantitative variables are expressed as median and interquartile range (IQR). Qualitative variables are presented as numbers and percent. Correlations between severity of sepsis surrogates and ONSD were analyzed using the Spearman’s rank correlation coefficient. Statistical significance was set at *p* < 0.05.

## 3. Results

The study group comprised 10 patients, five males and five females, at median age of 65 (IQR 50–78) years. The medians of APACHE II, SAPS II, and SOFA scores on admission were 22 (IQR 17–24), 52 (IQR 39.5–66), and 9 (IQR 8.5–12) points, respectively. Basic demographic, clinical, and laboratory parameters on ICU admission are presented in Table 1.

**Figure 1:**
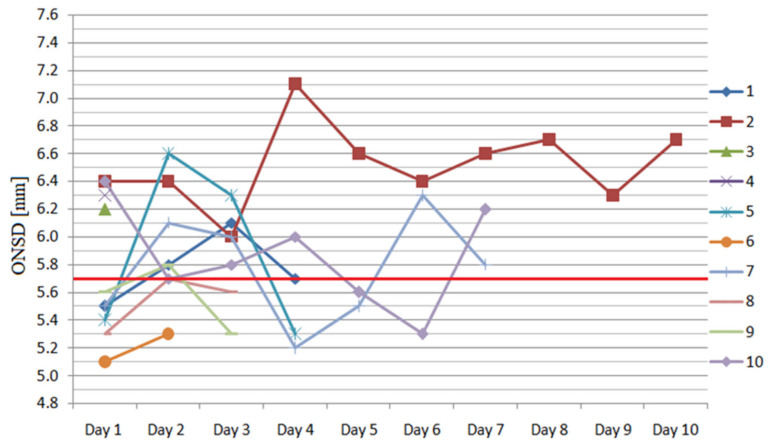
The consecutive measurements of the right eyeball optic nerve sheath diameter (ONSD) during the study period (red transverse line shows the cut-off value).

**Figure 2:**
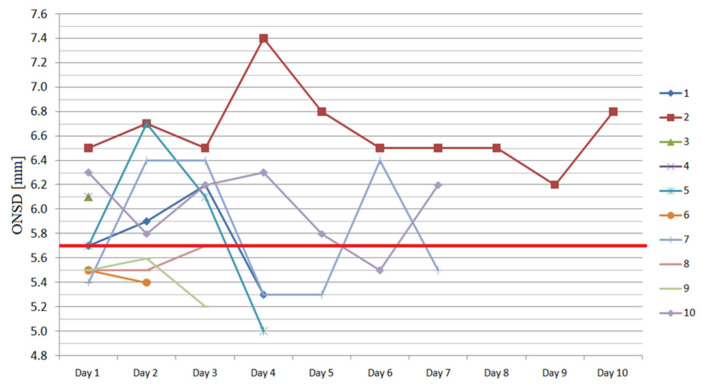
The consecutive measurements of the left eyeball ONSD during the study period (red transverse line shows the cut-off value).

The consecutive measurements of ONSD for each eyeball are presented in Figure 1 and Figure 2. On day 1, the medians of right and left ONSD were 5.56 (IQR 5.35–6.30) mm and 5.68 (IQR 5.50–6.10) mm, respectively, and four subjects had bilaterally elevated ONSD. Forty-nine out of the total 80 measurements performed (61%) exceeded 5.7 mm during the study period.

We found no correlation between ONSDs and CRP concentrations (right: R = −0.60–0.40, *p* = 0.28–0.60; left: R = −0.67–0.87, *p* = 0.22–0.8), highest daily lactate (right: R = −0.55–0.50, *p* = 0.17–0.82; left: R = −0.81–0.86, *p* = 0.05-0.82) or SOFA (right: R = −0.13–0.63, *p* = 0.33–0.88, left: R = −0.24–0.63, *p* = 0.36–0.91) during the study period.

## 4. Discussion

The aim of our study was to analyze ONSD in the population of septic shock patients and assess its potential role in screening for SABD. We found that 40% of subjects had ONSD above the cut-off point of 5.7 mm on the first day of observation, and ONSD fluctuated around upper limit during observation.

There is no specific reference range for ONSD. Different authors came out with different cut-off points. Some authors postulated lower cut-off values for ONSD (5.05–5.5 mm) [17,18,19]. By accepting slightly more restrictive cut-off value proposed by Lee (i.e., 5.5 mm), 70% of our patients on the first day of observation would have had ONSD above the upper limit. There are also some studies describing variable cut-off points for ONSD in different ethnic groups. Wang et al. set the optimal ONSD cut-off value for Chinese population at 4.1 mm, whereas Jeon et al. for Korean population at 5.6 mm [20,21]. Although there is no widely accepted cut-off value for ONSD, it has potential benefits as a surrogate of intracranial pressure. Some authors suggest using a ratio of ONSD to eyeball transverse diameter (ETD) to overcome the limitations associated with an optimal cut-off value [22]. The ratio ONSD/ETD takes into consideration anatomical differences among patients, the value above 0.25 is suggestive of intracranial hypertension. Optic nerve sheath diameter measurement is simple, non-invasive, safe, fast, and inexpensive [23]. Sepsis-associated brain dysfunction (SABD) is a common organ insufficiency in septic patients. It is considered an independent prognostic factor of increased mortality [24]. Pathophysiology of SABD is complicated, and one of the postulated factors is brain edema. The central nervous system is prone to various extra-cerebral factors, which is why dysfunction of the brain is often present in septic patients [25]. An important aspect of central nervous system homeostasis is a properly functioning blood-brain barrier. Patients with septic shock have an increased permeability of the blood-brain barrier due to inflammation (e.g., TNF-α). It causes an inflow of water and plasma proteins into the subarachnoid space causing an increase in ICP. Additionally, loss of cerebral blood flow autoregulation, frequently present in septic patients, aggravates the problem [26,27]. Salahuddin et al. found that coma in patients with non-traumatic radiographic cerebral edema was frequently due to septic encephalopathy [12]. Along with other organ systems monitored in septic patients, it is important to monitor the central nervous system for signs of dysfunction. As it is a non-invasive, quick, and widely accessible method, ONSD gives users the opportunity for early identification (screening) of the first signs of potential brain dysfunction in sepsis. Measurement of ONSD also provides an opportunity for continuous, non-invasive monitoring of brain edema. By implementing ONSD as an element of monitoring armamentarium in septic patients, the individual goal for arterial blood pressure and other measures to optimize cerebral perfusion pressure might be decided on. These might optimize cerebral perfusion pressure in septic shock patients. Whether implementing ONSD as a monitoring modality in septic patients is clinically beneficial remains unknown.

Patients diagnosed with septic shock have borderline/mildly elevated optic nerve sheath diameter (ONSD), which might be a sign of sepsis-associated brain dysfunction and marker of endothelial dysfunction. ONSD monitoring might be used for setting individual goals for arterial blood pressure in order to optimize cerebral perfusion in these patients.

### Study Limitations

The study group was relatively small, but this was a preliminary study. We did not take account of some factors having potential impact on ONSD (e.g., ventilator settings). We did not measure the ONSD/ETD ratio, which might have been a more objective measurement [18,28]. Increased ONSD in our study was only suggestive of SABD, as we did not measure brain injury biomarkers or perform other confirmatory tests (EEG, evoked potentials, computed tomography, magnetic resonance). Although there were different causes of sepsis in our study, there is no evidence that the source of infection influences the intensity/exacerbation of SABD. Other causes of elevated ONSD in our study are possible; however, none of our patients was diagnosed with a neuro-infection. Analyzing the association between ONSD (a metric measurement) and SOFA (an ordinal scale) was also a limitation.

## 5. Conclusions

ONSD above the cut-off value is common in septic shock patients. It might have a potential role for screening for SABD; however, further research is needed to investigate its exact role.

## Figures and Tables

**Table 1:** Demographic, clinical and laboratory parameters of patients on the first day of observation.

Patient	Sex (M/F)	Age (Years)	APACHE II ^1^	SAPS II ^2^	SOFA ^3^	CRP ^4^ (mg L^−^^1^)	PCT ^5^ (ng mL^−^^1^)	Lactate (mmol L^−^^1^)	ONSD ^6^ R (mm)	ONSD ^6^ L (mm)
1	F	83	26	74	14	182.8	3.07	3.55	5.5	5.7
2	M	55	21	44	12	204.8	1.62	2.00	6.4	6.5
3	M	63	22	58	7	109.5	1.41	2.60	6.2	6.1
4	F	69	35	61	15	470.8	49.51	2.66	6.3	6.1
5	F	82	18	46	9	102.4	2.45	3.99	5.4	5.6
6	M	65	22	52	10	327.7	3.54	16.03	5.1	5.5
7	M	73	42	85	17	146.7	3.24	1.99	5.5	5.4
8	F	40	16	27	9	331.0	54.57	5.14	5.3	5.5
9	F	82	13	58	9	194.3	60.80	7.59	5.6	5.5
10	M	35	15	33	8	72.5	0.94	3.48	6.4	6.3

^1^ Acute Physiology and Chronic Health Evaluation II [points], ^2^ Simplified Acute Physiology Score II [points], ^3^ Sequential Organ Failure Assessment [points], ^4^ C-reactive protein, ^5^ procalcitonin, ^6^ optic nerve sheath diameter [R—right; L—left].
